# Prevalence of postprandial hypotension in older adults: a systematic review and meta-analysis

**DOI:** 10.1093/ageing/afae022

**Published:** 2024-02-26

**Authors:** Lei Huang, Sheyu Li, Xiaofeng Xie, Xiaoli Huang, Lily Dongxia Xiao, Ying Zou, Wenyi Jiang, Fengying Zhang

**Affiliations:** West China School of Nursing/Nursing Key Laboratory of Sichuan Province/Innovation Center of Nursing Research/West China Hospital, Sichuan University, 37, Guoxue Alley, Chengdu, Sichuan, 610041, China; Department of Endocrinology and Metabolism, West China Hospital, Sichuan University, Chengdu, Sichuan 610041, China; West China School of Nursing/Nursing Key Laboratory of Sichuan Province/Innovation Center of Nursing Research/West China Hospital, Sichuan University, 37, Guoxue Alley, Chengdu, Sichuan, 610041, China; Center of Gerontology and Geriatrics, National Clinical Research Center for Geriatrics, West China Hospital, Sichuan University, Chengdu, Sichuan Province 610041, China; College of Nursing and Health Sciences, Flinders University, Adelaide, Australia; West China School of Nursing/Nursing Key Laboratory of Sichuan Province/Innovation Center of Nursing Research/West China Hospital, Sichuan University, 37, Guoxue Alley, Chengdu, Sichuan, 610041, China; West China School of Nursing/Nursing Key Laboratory of Sichuan Province/Innovation Center of Nursing Research/West China Hospital, Sichuan University, 37, Guoxue Alley, Chengdu, Sichuan, 610041, China; West China School of Nursing/Nursing Key Laboratory of Sichuan Province/Innovation Center of Nursing Research/West China Hospital, Sichuan University, 37, Guoxue Alley, Chengdu, Sichuan, 610041, China

**Keywords:** older adults, postprandial hypotension, prevalence, systematic review

## Abstract

**Background:**

Older adults with postprandial hypotension (PPH) increase susceptibility to falls, syncope, stroke, acute cardiovascular diseases and even death. However, the prevalence of this condition varies significantly across studies. We aimed to determine the prevalence of PPH in older adults.

**Methods:**

Web of Science, PubMed, Cochrane Library, Embase and CINAHL were searched from their inception until February 2023. Search terms included ‘postprandial period’, ‘hypotension’ and ‘postprandial hypotension’. Eligible studies were assessed using the Joanna Briggs Institute tool. Meta-analyses were performed among similar selected studies.

**Results:**

Thirteen eligible studies were included, and data from 3,021 participants were pooled. The meta-analysis revealed a PPH prevalence of 40.5% [95% confidence interval (CI): 0.290–0.519] in older adults, and this was prevalent in the community (32.8%, 95% CI: 0.078–0.647, *n* = 1,594), long-term healthcare facility (39.4%, 95% CI: 0.254–0.610, *n* = 1,062) and geriatrics department of hospitals (49.3%, 95% CI: 0.357–0.630, *n* = 365). The pooled results showed significant heterogeneity (*I*^2^ > 90%), partially related to the different ages, sex, pre-prandial systolic blood pressure levels of participants, or the different criteria and methodology used to diagnose PPH.

**Conclusions:**

PPH is a prevalent condition in older adults. Further research is needed to confirm this result, and priority should be given to establishing international consensus on PPH diagnostic criteria and designing its diagnostic procedure.

## Key Points

- Older adults with postprandial hypotension (PPH) are predisposed to falls, syncope, emerging cardiovascular diseases, stroke and even mortality.
- PPH affects 40.5% (95% confidence interval: 0.290–0.519) of older adults.
- Establishing international consensus on PPH diagnostic criteria and designing its diagnostic procedure should be priorities.

## Background

Postprandial hypotension (PPH) is a health condition characterised by a meal-related reduction in blood pressure (BP). It probably reflects the complex interactions of multiple factors, involving the ageing process and specific diseases that impair the baroreflex, high carbohydrate meals, abnormal gastric emptying, postprandial stagnation of visceral blood and a significant release of vasoactive peptides after eating [1–3]. These factors disturb the homeostasis of BP regulation, causing the excursion of postprandial BP. Although a recent study shows that PPH is not associated with postprandial impairment of attentive functions for older inpatients [4], it can alter normal gait and cause falls [5, 6], and is an important feature suggestive of a syncope diagnosis [6, 7]. A recent systematic review has further identified that older adults with PPH are predisposed to emerging cardiovascular diseases, stroke and even mortality [8].

PPH is thus positively related to various adverse events for older individuals. However, its prevalence in this population is still unclear, as current studies report a wide prevalence of PPH, ranging from 9.0% to 91.0% [1, 9–11], and existing systematic review only demonstrates that PPH is prone to adults with neurological disorders (e.g. diabetes, Parkinson’s disease and Alzheimer’s disease) [12]. In addition, the symptom of PPH lacks specificity, presenting with sleepiness, fatigue or nausea [13, 14], thus leading to PPH not being detected in older adults. The methodology of diagnosing PPH should include measuring BP by ambulatory blood pressure monitoring (ABPM) or manually monitoring BP at intervals of 10–30 min until 2 h after the start of eating [1, 15]. There are few routine screens for PPH in clinical settings owing to the time-consuming procedure, strained medical care human resources and limited availability of ABPM devices [15–17]. Therefore, PPH has not received enough attention among older adults.

We performed this systematic review to ascertain the prevalence of PPH in older adults from communities, long-term healthcare facilities and hospitals. Given the detrimental effects of PPH on older adults and the rapidly ageing population worldwide, determining reliable estimates of PPH prevalence among older adults is imperative to raise awareness among health workers; facilitate evidence-based prevention, treatment and care; and importantly, promote health in older adults [18].

## Methods

### Protocol and registration

The protocol for this review was registered at PROSPERO (CRD42022362505) and adhered to the Preferred Reporting Items for Systematic Review and Meta-analysis guidelines [19].

### Inclusion criteria

Published papers written in English that met the following aspects were incorporated: (i) *Population*—the mean or median age of participants was ≥60 years. (ii) *Condition*—studies were included if they reported the prevalence of PPH or it could be calculated based on the reported data, and the following diagnostic criteria were used. These comprised a decrease in postprandial systolic blood pressure (SBP) of ≥20 mmHg relative to the pre-prandial SBP or the postprandial SBP declining to ≤90 mmHg when the pre-prandial SBP was ≥100 mmHg within 120 min after the start of a meal [1]. In addition, if the prevalence of PPH was reported on different days, the first point in time would prevail [20]. (iii) *Context*—communities, long-term healthcare facilities and hospitals. (iv) *Study design*—cross-sectional or cohort design.

### Exclusion criteria

Studies partially or completely demonstrating the following characteristics were excluded: (i) participants only belonging to a subgroup of older adults, such as ageing men and older adults with hypertension, falls or syncope; (ii) studies that have adopted a cross-sectional design with a sample size of <50 cases [21]. For cohort studies, only those with a group sample size of ≥50 cases were included, and their baseline data were extracted.

### Literature retrieval and screening

Two independent reviewers searched and screened for related studies, and a third independent reviewer resolved their disagreements. Web of Science, PubMed, Cochrane Library, Embase (Elsevier) and CINAHL (EBSCO) were retrieved from their inception until February 2023. The search strategy involved combining MeSH terms with entry terms, along with using keywords that encompassed ‘postprandial period’, ‘hypotension’ and ‘postprandial hypotension’ (Table S1, available in *Age and Ageing* online). EndNote X9 was used to remove duplicate entries. For studies that potentially complied with the inclusion criteria, their full texts were carefully and independently assessed. References cited by authors of the included studies, reviews, guidelines, opinions, letters and book chapters were searched for eligible full texts [18].

### Risk-of-bias assessment

The risk of bias (ROB) of incorporated studies were evaluated by two independent reviewers, and their disagreements were resolved by a third independent reviewer. The Joanna Briggs Institute (JBI) critical appraisal checklist for studies reporting prevalence data was used to rate the methodological quality of included studies (Table S2, available in *Age and Ageing* online). The result of each question was the answer of ‘Yes’, ‘No’ or ‘Not applicable’. Only when a question was answered adequately did the study receive a ‘Yes’ response. The evaluation results of each study were presented as an adherence rate, which was calculated by determining the number of questions with the answer ‘Yes’ among the total number of questions presented [18, 22].

### Data extraction

The following data from the included studies were extracted by two independent reviewers using a standardised data sheet: (i) *Study characteristics*—author, publication year, country, setting, design and sample. (ii) *Population features—*age, sex, pre-prandial BP and the most common cardiovascular disease among participants. (iii) *Criteria for diagnosing PPH.* (iv) *Methodology of diagnosing PPH—*including the preparations (e.g. withdraw medications, fast, and prohibition of caffeine, alcohol or smoking) and procedures [e.g. monitoring period (e.g. breakfast, lunch, dinner, or across multiple meals), positions, determining the pre-prandial BP, test meal, and time intervals and duration of measuring postprandial BP] used to diagnose PPH, and the BP measuring device and its principle. *Outcome*—prevalence of PPH or related raw data.

### Statistical analyses

When three or more studies were similar, a meta-analysis was performed. Data conversion was carried out to ensure they complied with the normal distribution. Cochran’s *Q* test was utilised to examine heterogeneity among the selected studies, and the *I*^2^ statistic served as the measure of heterogeneity. The threshold values of the *I*^2^ statistic were 25%, 50% and 75%, representing low, moderate and high heterogeneity, respectively. Owing to the significant heterogeneity among studies reporting prevalence data, a random-effects model was employed to synthesise the data. Subgroup analyses were performed to investigate the source of heterogeneity, and the corresponding group criteria included mean age, sex, pre-prandial SBP, diagnostic criteria of PPH, withdrawal medications, monitoring period, and the time interval and duration of measuring postprandial BP. Univariate meta-regression analyses were carried out to investigate the source of heterogeneity, and adjusted covariates included settings, mean age, proportion of females and hypertension, and diagnostic criteria for PPH. Funnel plots and Egger’s test detected publication bias. A *P* value <0.05 was deemed statistically significant [18]. All statistical analyses were performed using R version 4.2.2 (https://cran.r-project.org/).

## Results

### Selection process

**Figure 1:**
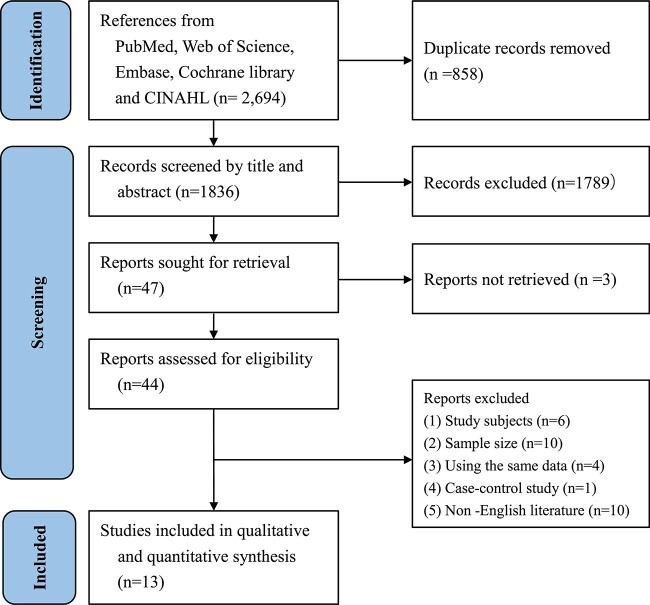
PRISMA flow diagram.

Of 2,694 studies retrieved, 47 were included for full-text evaluation after removing the duplicates and examining their titles and abstracts. The full text of three of these studies could not be retrieved, and finally, 13 studies met the inclusion criteria and were incorporated (Figure 1).

### Study characteristics

The included studies were conducted in the following countries: South Korea [23, 24], France [25, 26], Australia [27, 28], Japan [9, 29], the USA [30, 31], Belgium [10], Mexico [11] and the Netherlands [14]. Papers were published between 1991 and 2020. Nine and four studies used cross-sectional and cohort design, respectively (Table 1). Participants were enlisted from diverse settings: three studies recruited participants from communities [9, 23, 27]; four from long-term healthcare facilities [24, 28, 30, 31]; five from hospitals, specifically from an acute geriatric department [10], geriatric rehabilitation centre [25] and geriatric department [14, 26, 29]; and the remaining from retirement homes and communities [11]. The inclusion and exclusion criteria varied across the included studies [9–11, 14, 23–31], and the sample sizes fluctuated between 50 and 1,308 cases. In total, 3,021 participants were incorporated in this review.

**Table 1:** Basic characteristics of included studies

Author, year, country	Design, sample	Age (y), *F* (%)	Pre-prandial BP (SBP/DBP, mmHg), CVD (%)	Prevalence (%)
**Community**				
Jang, A., 2020 [23], South Korea	Co, 94	73.1 ± 4.8, 84.0	128.6/75.2, HT (50.0)	50.0
Trahair *et al*., 2015 [27], Australia	CS, 86	71.0 ± 0.5, 53.4	122.3/69.0, U	12.8
Tabara *et al*., 2014 [9], Japan	CS, 1,308	65.2 ± 9.1, 60.7	126.0/72.0, U	9.0
**Long-term healthcare facility**				
Son *et al.*, 2012 [24], South Korea	CS, 121	81.7 ± 7.0, 79.3	136.8/74.1, HT (49.6)	32.2
Fisher *et al*., 2005 [28], Australia	Co, 179	83.2 ± 7.0, 80.0	156.3–156.6/82.3–84.6, HT (47.5)	38.0
Aronow *et al*., 1994 [30], USA	Co, 499	80.0 ± 9.0, 71.0	135.0/78.0, CAD (42.5)	23.6
Vaitkevicius *et al*., 1991 [31], USA	Co, 113	78.0 ± 9.0, 73.0	140.0/73.0, CD (39.8)	36.3
**Geriatrics’ department of hospital**				
Schoevaerdts *et al*., 2019 [10], Belgium	CS, 76	84.9 ± 4.9, 58.0	U/U, HT (66.0)	46.0
Abbas *et al*., 2018 [25], France	CS, 104	86.0, 67.3	U/U, HT (86.5)	37.5
Vloet *et al*., 2005 [14], The Netherlands	CS, 85	80.0 ± 1.0 48.2	147.0/82.0, CD (57.6)	67.1
Puisieux *et al*., 2002 [26], France	CS, 50	83.0 ± 8.0 84.0	146.0/81.5^a^^,^^b^, HT (60.0)	64.0
Teramoto *et al*., 1997 [29], Japan	CS, 50	73.9–74.3, U	132.0–142.0/77.0–82.0, HT (6.0)	32.0
**Mixed population**				
Asensio *et al*., 2015 [11], Mexico	CS, 256	78.1 ± 8.8, 76.2	U/U, HT (45.7)	80.1

y, year; F, female; BP, blood pressure; SBP, systolic blood pressure; DBP, diastolic blood pressure; CVD, cardiovascular diseases; Co, cohort; HT, hypertension; CS, cross-sectional; U, unreported; CAD, coronary artery disease; CD, cardiac disorders.

^a^Data were extracted by Image Data Tools named Origin 2022.

^b^When the research reported pre-prandial BP values at different time points, the higher time point should prevail.

### Population features

The mean age of participants ranged between 65.2 and 86.0 years (Table 1). Generally, more women were recruited. Ten included studies reported that the pre-prandial BP level of participants ranged from 122.3 to 156.6 mmHg for pre-prandial SBP and 69.0 to 84.6 mmHg for pre-prandial diastolic blood pressure (DBP) [9, 14, 23, 24, 26–31], while the remaining studies did not report this aspect [10, 11, 25]. Eight and three included studies reported that the most common cardiovascular disease among participants were hypertension and cardiac disorders, respectively. However, the remaining did not report this aspect (Table 1).

### Diagnostic criteria of PPH

Thirteen included studies utilised 10 diverse diagnostic criteria for PPH. We divided them into three categories, within 120 min after eating: (i) *Type I:* the maximum reduction in postprandial SBP ≥20 mmHg accounted for 11/13 [9, 14, 23–31]; (ii) *Type II:* postprandial SBP declined to ≤90 mmHg if pre-prandial SBP was ≥100 mmHg [10]; (iii) *Type III:* a reduction in postprandial SBP of ≥20 mmHg and DBP of ≥10 mmHg compared to pre-prandial BP [11].

### Methodology of diagnosing PPH

The methodology of diagnosing PPH varied significantly between studies (Table 2): (i) *Preparations:* Before diagnosing PPH, seven studies asked participants to withdraw from any medication use [14, 23, 27–31], three did not [24–26] and the remaining did not report this aspect [9–11]. Except for six studies that required participants to fast, maintaining a duration of at least 4 h was required [14, 23, 26–29], but seven studies did not provide this information [9–11, 24, 25, 30, 31]. For additional details, see Table S3, available in *Age and Ageing* online. (ii) *Procedures:* Regarding the BP monitoring period, five studies selected around lunch [9, 24, 25, 30, 31], four around breakfast [14, 27–29], one around breakfast and lunch [11], one around breakfast and dinner [26], and the remaining studies did not report this information [10, 23]. Nine studies adopted a sitting position [10, 14, 23–26, 28–30], three did not report this aspect [9, 11, 27] and the remaining did not standardise the position [31]. The included studies employed diverse techniques to determine the pre-meal BP [9–11, 14, 23–31]. Five studies utilised a standard test meal, including meal replacements (e.g. 300 mL glucose solution containing 75 g of glucose, Table S4, available in *Age and Ageing* online) or common food (e.g. 210 g rice, 100 g soup and 70 g side dishes) [14, 23, 26, 27, 29], while the remaining studies did not standardise the test meal [9–11, 24, 25, 28, 30, 31]. The time interval and duration of postprandial BP measurement were 30 and 30 min [9], 5–60 and 60 min [28, 29], 10–15 and 90 min [10, 11, 14, 24, 25, 31], and 5–45 and 120 min [23, 26, 27, 30], respectively. (iii) *Device and principles:* The included studies usually utilised BP devices with the upper arm sleeve [9, 10, 14, 23, 24, 26–28, 30, 31], such as automated sphygmomanometers [23, 31], mercury sphygmomanometers [24, 30] and ABPM [14, 28], and the oscillometric method was the most widely used for measuring BP [9, 10, 14, 23, 26–29, 31].

**Table 2:** Methods of diagnosing postprandial hypotension

Author, year	Diagnostic criteria	Withdraw medications/fast	Procedure (monitoring period/sitting position/standard test meal/the time interval and duration of measuring postprandial BP, min)	BP measuring device with upper arm sleeve/principle
Jang, 2020 [23]	①	Y/Y	U/Y/Y/15/120	Y/OM
Schoevaerdts *et al*., 2019 [10]	②③	U/U	U/Y/N/15/90	Y/OM
Abbas *et al*., 2018 [25]	④	N/U	L/Y/N/15/90	U/U
Asensio *et al*., 2015 [11]	⑤	U/U	B&L/U/N/10/90	U/U
Trahair *et al*., 2015 [27]	④	Y/Y	B/U/Y/5/120	Y/OM
Tabara *et al*., 2014 [9]	⑥	U/U	L/U/N/30/30	Y/OM
Son *et al*., 2012 [24]	②	N/U	L/Y/N/15/90	Y/AM
Fisher *et al*., 2005 [28]	⑦	Y/Y	B/Y/N/60/60	Y/OM
Vloet *et al*., 2005 [14]	④	Y/Y	B/Y/Y/15/90	Y/OM
Puisieux *et al*., 2002 [26]	⑧	N/Y	B&D/Y/Y/15/120	Y/OM
Teramoto *et al*., 1997 [29]	⑨	Y/Y	B/Y/Y/5/60	U/OM
Aronow *et al*., 1994 [30]	①	Y/U	L/Y/N/15~45/120	Y/AM
Vaitkevicius *et al*., 1991 [31]	⑩	Y/U	L/U#/N/15/90	Y/OM

BP, blood pressure; min, minute; Y, yes; U, unreported; OM, oscillographic method; N, no; L, lunch; B, breakfast; AM, auscultatory method; U#, unstandardized.① PPH was defined as a decrease in SBP of ≥20 mmHg within 120 min after eating; ② PPH was defined as a drop of SBP of >20 mmHg within 120 min after eating; ③ PPH was defined as a drop of SBP ≤90 mmHg if pre-prandial SBP was ≥100 mmHg within 120 min after eating; ④ PPH was defined as a decrease in SBP of ≥20 mmHg within 90 min after eating; ⑤ PPH was defined as a reduction of ≥20 mmHg for SBP and ≥10 mmHg for DBP within 90 min after eating, in both cases compared to pre-prandial BP; ⑥ PPH was defined as a decrease in SBP of >20 mmHg within 30 min after eating; ⑦ PPH was defined as a decrease in SBP of ≥20 mmHg within 60 min after eating; ⑧ PPH was defined as a SBP decline ≥20 mm Hg within 120 min of the start of a meal; ⑨ PPH was defined as a decrease in SBP of >20 mmHg within 60 min after eating; ⑩ PPH was defined as a drop of SBP of >20 mmHg within 90 min after eating.

### Results of ROB assessments

The incorporated studies had moderate to high ROB because their adherence rates ranged from 22.2% to 55.6% [18, 22]. The main sources of ROB were selective bias, small sample size, unclear reports of participants and settings, and lack of validated criteria and standardised procedures for diagnosing PPH (Table S2, available in *Age and Ageing* online).

### Prevalence of PPH in older adults

#### Community-dwelling older adults

Four studies reported the prevalence of PPH in community-dwelling older adults, which ranged from 9.0% to 69.8% [9, 11, 23, 27]. The pooled prevalence in older adults aged 65.2 to 76.5 years was 32.8% (95% CI: 0.078–0.647, *I*^2^ = 98.8%, *P* < 0.05, *n* = 1,594, Figure S1, available in *Age and Ageing* online). Specifically, the prevalence was 9.0% (95% CI: 0.075–0.107) in older adults aged 65.2 years (*n* = 1,308) [9] and 36.6% (95% CI: 0.136–0.984, *I*^2^ = 94.9%, *P* < 0.05; *n* = 286) in older adults aged ~70.0 years [11, 23, 27].

#### Long-term healthcare facility residents

Five studies reported the prevalence of PPH in older adults living in long-term healthcare facilities, which ranged from 23.6% to 87.3% [11, 24, 28, 30, 31]. The pooled prevalence in older adults aged 78.0 to 83.2 years was 39.4% (95% CI: 0.254–0.610, *I*^2^ = 98.8%, *P* < 0.05; *n* = 1,062, Figure S2, available in *Age and Ageing* online). Specifically, the prevalence was 36.3–87.3% in older adults aged ~70.0 years [11, 31] and 30.7% (95% CI: 0.224–0.397, *I*^2^ = 85.8%, *P* < 0.05; *n* = 799) in those aged ~80.0 years [24, 28, 30].

#### Geriatric patients

The remaining five studies reported the prevalence of PPH in older adults from the geriatrics department of hospitals, which ranged from 32.0% to 67.1% [10, 14, 25, 26, 29]. The pooled prevalence in older adults aged 73.9 to 86.0 years was 49.3% (95% CI: 0.357–0.630, *I*^2^ = 85.4%, *P* < 0.05; *n* = 365, Figure S3, available in *Age and Ageing* online). Specifically, the prevalence was 32.0% for older adults aged ~70.0 years [29] and 52.8% (95% CI: 0.403–0.692, *I*^2^ = 84.7%, *P* < 0.05; *n* = 315) for those aged ~80.0 years [10, 14, 25, 26].

**Figure 2:**
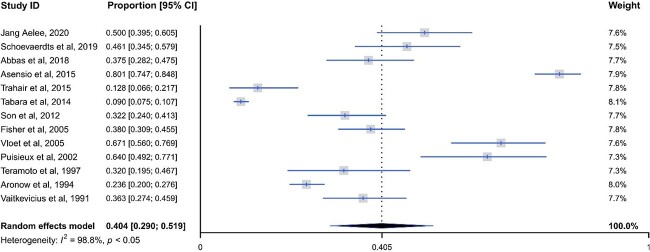
Forest plot of studies reporting the prevalence of postprandial hypotension in older adults.

Based on the 13 included studies, the pooled prevalence in older adults aged 65.2 to 86.0 years was 40.5% (95% CI: 0.290–0.519, *I*^2^ = 98.8%, *P* < 0.05; *n* = 3,021, Figure 2) with high heterogeneity. The univariate meta-regression analysis revealed that settings of recruiting participants could account for 50.8% of the heterogeneity (*P* < 0.05, Table S5, available in *Age and Ageing* online), that the type of diagnostic criteria for PPH could account for 24.7% (*P* < 0.05) and that the mean age of participants could account for 16.9% but with no statistical significance (*P* = 0.08). Funnel plot asymmetry and the result of Egger’s test (*t* = 3.01, *P* = 0.01, Figure S4, available in *Age and Ageing* online) did not represent existing public bias, as only observational studies were included in this systematic review. Still, most included studies had a small sample size [32].

### Subgroup analysis

Subgroup analysis was conducted to further reveal the source of heterogeneity or characteristics of PPH based on the following aspects (Table 3): (i) *Population features*: The pooled prevalence of PPH was 9.0%, 37.2% and 41.6% in older adults aged ~60.0, 70.0 and 80.0 years, respectively. The PPH prevalence was 52.3% for older females and 54.5% for older males. When pre-prandial SBP was 120–139 mmHg, the pooled prevalence was 22.5%; however, it increased to 50.9% when pre-prandial SBP was 140–159 mmHg. (ii) *Diagnostic criteria for PPH*: When researchers adapted type I, type II and type III diagnostic criteria for PPH, the pooled prevalence of PPH was 35.2%, 46.1% and 80.1%, respectively. (iii) *Preparations:* The pooled prevalence of PPH was 35.6% when withdrawing participants’ daily medications, while it was 43.8% for participants taking daily medications as usual. (iv) *Procedures*: The pooled prevalence of PPH was 37.4%, 27.1%, 32.0% and 64.0–80.1% when BP was measured around breakfast, lunch, dinner and across multiple meals, respectively. The PPH prevalence was 32.7% when the test meal was not standardised and 44.2% when it was. The pooled prevalence was 9.0%, 32.0–38.0%, 47.8% and 37.1% when the time interval between BP measurements and duration of postprandial BP measurement was 30 and 30 min, 60 and 60 min, 10–15 and 90 min, and 5–45 and 120 min, respectively. (v) *Prevalence of symptomatic PPH*: Three studies reported the prevalence of symptomatic PPH, which was checked by investigators during the PPH diagnosis in hospital scenarios (Table S6, available in *Age and Ageing* online), among older adults aged ~80 years, which ranged from 12.5% to 43.5% [10, 14, 25]. The pooled prevalence of symptomatic PPH was 22.2%. However, substantial heterogeneity should be considered (*I*^2^ = 86.6–99.0%, *P* < 0.05, Table 3).

**Table 3:** Results of subgroup analysis

Grouping criteria	Participants	Prevalence (%, 95% CI)	*I* ^2^ (%)
Mean age (y)			
60–69 [9]	1,308	9.0 (7.5–10.7)	NA
70–79 [11, 23, 27, 29, 31]	599	37.2 (21.0–65.9)	96.3
80–89 [10, 14, 24–26, 28, 30]	1,114	41.6 (31.4–55.1)	94.6
Sex			
Male [10, 11, 23, 25]	142	54.5 (32.8–75.3)	87.7
Female [10, 11, 23, 25]	388	52.3 (37.5–72.8)	93.4
Pre-prandial SBP (mmHg)			
120–139 [9, 23, 24, 27, 30]	2,108	22.5 (12.3–37.6)	97.3
140–159 [14, 26, 28, 31]	427	50.9 (34.7–66.9)	90.4
Type of diagnostic criteria for PPH			
Type I [9, 14, 23–31]	2,689	35.2 (24.2–47.0)	97.3
Type II [10]	76	46.1 (34.6–57.9)	NA
Type III [11]	256	80.1 (74.7–84.8)	NA
Withdraw medications			
Yes [14, 23, 27–31]	1,106	35.6 (23.9–49.3)	93.0
No [24–26]	275	43.8 (25.8–62.6)	86.6
BP monitoring period			
Around breakfast [14, 26–29]	450	37.4 (20.1–54.8)	94.9
Around lunch [9, 24, 25, 30, 31]	2,145	27.1 (16.4–37.8)	96.9
Around dinner [26]	50	32.0 (19.5–46.7)	NA
Across multiple meals [11, 26]	306	64.0–80.1	NA
Used standard test meal			
Yes [14, 23, 26, 27, 29]	365	44.2 (23.8–65.7)	94.6
No [9–11, 24, 25, 28, 30, 31]	2,656	32.7 (21.1–50.7)	99.0
Time interval and duration of monitoring postprandial BP			
30/30 [9]	1,308	9.0 (7.5–10.7)	NA
5–60/60 [28, 29]	229	32.0–38.0	NA
10–15/90 [10, 11, 14, 24, 25, 31]	755	47.8 (35.3–64.5)	95.7
5–45/120 [23, 26, 27, 30]	729	37.1 (14.2–59.9)	95.6
Symptomatic PPH [10, 14, 25]	265	22.2 (6.7–43.1)	92.7

y, year; NA, not applicable; SBP, systolic blood pressure; PPH, postprandial hypotension; Type I, PPH was defined as a decrease in SBP of ≥20 mmHg within 120 min after eating; Type II, PPH was defined as a drop of SBP of >20 mmHg or postprandial SBP ≤90 mmHg if pre-prandial SBP was ≥100 mmHg within 120 min after eating; Type III, PPH was defined as a reduction of ≥20 mmHg for SBP and ≥10 mmHg for DBP within 90 min after eating, in both cases compared to pre-prandial BP.

## Discussion

This study demonstrated that PPH affected 40.5% of older adults and was different from previous literature reviews, which reported that the prevalence of PPH was 20.0–91.0% [1]. We have updated the results qualitatively synthesised by the study published in 2014 [1]. Furthermore, we assessed the ROB of the included studies, qualitatively and quantitatively synthesised the pertinent data, quantified the studies’ heterogeneity and performed various explorations. Thus, under the guidance of the evidence-based medicine methodology, we first synthesised the existing evidence about the prevalence of PPH in older adults. However, our pooled result should be interpreted cautiously considering that (i) the involved studies had moderate to high ROB, which partially came from selective bias owing to convenience sampling, reducing the representation of the population. (ii) A small sample size existed in most incorporated studies, leading to the reported results often being overestimated [32]. (iii) The pooled result showed significant heterogeneity, likely deriving from participants recruited from different settings, being of different ages and sexes, with varied pre-prandial SBP levels [33–36]. However, the heterogeneity was not surprising as we have focused on the population widely distributed with various characteristics. The pooled result is likely related to the real world. Therefore, although more studies with large sample sizes and rigorous designs should be conducted to validate the result, our review is sufficient to inform health workers of the potentially high prevalence of PPH among older adults [1] and the need to develop more resources to reduce the disease burden [8].

Our review found that the prevalence of PPH in older adults was 32.8% in communities, 39.4% in long-term healthcare facilities and 49.3% in geriatrics department of hospitals. A cohort study states that for geriatric patients, the incidence of PPH decreases from 27.3% to 9.1% as their illnesses resolve [36]. This phenomenon probably explained why our review found that PPH was predisposed to older patients compared with others. However, this study did not find any mechanism, such as the improvement of gastric dysmotility and cardiovascular autonomic dysfunction, that could explain it [36]. Given the generally suboptimal management strategies for this disease, more studies are needed to investigate factors that predict PPH.

This research indicated that lacking widely recognised criteria for diagnosing PPH increased the ROB and augmented the significant heterogeneity. Researchers have demonstrated that the meal-related reduction in SBP predisposed to falls, syncope, emerging cardiovascular diseases, stroke and even mortality [5–8]; however, the clinical relevance of the type III criterion remains unclear. Analogous to postprandial SBP decrease, multiple studies identified that postprandial DBP decline was a common phenomenon for older adults [10, 23, 24]. Hence, further research should be conducted to elucidate the pathophysiological mechanism and precise clinical significance of the multiple diagnostic criteria to establish international consensus on this clinical issue.

Our study found that the lack of a standard methodology for diagnosing PPH has increased the ROB and contributed to the high heterogeneity. Daily medications should not be halted during diagnosis, considering the ethics and real-world situations [37]; however, potentially inappropriate medications should be reviewed. The decision to prohibit foods, caffeine, alcohol or smoking should also be unified (Table S3, available in *Age and Ageing* online), given that these factors impact the BP [38] and increase between-study heterogeneity. Our review further indicates that PPH is more likely to occur after breakfast, which aligns with existing studies [33]; thus, PPH screening around breakfast is favourable [33]. The existing studies reported that adopting different positions and performing daily activities had no impact on the detection of PPH [39]; however, it is encouraged to use a consistent position during PPH diagnosis and report these details to minimise heterogeneity across studies. Given that BP continuously fluctuates, pre-prandial BP determined just before eating was probably a suitable method to increase the similarity between different studies [30, 40]. Previous studies suggest establishing the time interval and duration of postprandial BP monitoring based on the postprandial BP reduction trajectory induced by a certain meal replacement to ensure precise PPH detection [39, 41]. Noteworthy, few studies have investigated real-world homogeneity, clinical validity and utility of the meal replacement proposed by Jansen and Lipsitz or others and designed corresponding methods of diagnosing PPH [1]. Accounting for varied eating habits, the potential large scale of older adults affected by PPH and the limited availability of medical resources, validating and developing appropriate meal replacement and designing corresponding BP measurement methods should be considered to precisely and effectively diagnose PPH.

This review indicated that symptomatic PPH possibly affected 22.2% of older adults at an advanced age. Considering that it probably contributed to falls and syncope, cautiously defining symptomatic PPH and developing appropriate assessment tools should be a focus of future research to accurately and effectively identify it, eventually reducing the hazard of symptomatic PPH [10, 14, 15, 25].

Our research had some limitations. First, it had a certain degree of selection bias because we only included English literature published before February 2023. Moreover, some key information was lost because we did not acquire data from the original papers by contacting the authors. Furthermore, most of the involved studies did not report test meals in detail, which restricted reviewers from further exploring the heterogeneity.

## Conclusions

PPH is a prevalent condition in older adults. Further research is needed to confirm this result, and priority should be given to establishing international consensus on PPH diagnostic criteria and designing its diagnostic procedure.

## Supplementary Material

aa-23-1705-File002_afae022

## Data Availability

The data analysed in this research can be found in the public domain.
