# Supplementary material for: Prevalence of postprandial hypotension in older adults: a systematic review and meta-analysis
Source: Age Ageing. 2024 Feb 26;53(2):afae022. doi: 10.1093/ageing/afae022 (PMC10898335; doi:10.1093/ageing/afae022)
Supplement: aa-23-1705-File002_afae022 [file aa-23-1705-file002_afae022.docx]

**Table S1. Search strategy (PubMed)**

| **ID** | **Search** | **Hits** |
| --- | --- | --- |
| 1 | Postprandial Period[MeSH Terms] | 10,961 |
| 2 | ((((Period*, Postprandial[Title/Abstract]) OR (Postprandial Period*[Title/Abstract])) OR (Postcibal Period*[Title/Abstract])) OR (Period*, Postcibal[Title/Abstract])) OR (Postprandial state*[Title/Abstract]) | 13,092 |
| 3 | (Postprandial Period[MeSH Terms]) OR (((((Period*, Postprandial[Title/Abstract]) OR (Postprandial Period*[Title/Abstract])) OR (Postcibal Period*[Title/Abstract])) OR (Period*, Postcibal[Title/Abstract])) OR (Postprandial state*[Title/Abstract])) | 15,105 |
| 4 | Hypotension[MeSH Terms] | 29,757 |
| 5 | (((Vascular Hypotension[Title/Abstract]) OR (Low Blood Pressure[Title/Abstract])) OR (Blood Pressure, Low[Title/Abstract])) OR (Hypotension, Vascular[Title/Abstract]) | 2,401 |
| 6 | (Hypotension[MeSH Terms]) OR ((((Vascular Hypotension[Title/Abstract]) OR (Low Blood Pressure[Title/Abstract])) OR (Blood Pressure, Low[Title/Abstract])) OR (Hypotension, Vascular[Title/Abstract])) | 31,646 |
| 7 | ((Postprandial Period[MeSH Terms]) OR (((((Period*, Postprandial[Title/Abstract]) OR (Postprandial Period*[Title/Abstract])) OR (Postcibal Period*[Title/Abstract])) OR (Period*, Postcibal[Title/Abstract])) OR (Postprandial state*[Title/Abstract]))) AND ((Hypotension[MeSH Terms]) OR ((((Vascular Hypotension[Title/Abstract]) OR (Low Blood Pressure[Title/Abstract])) OR (Blood Pressure, Low[Title/Abstract])) OR (Hypotension, Vascular[Title/Abstract]))) | 176 |
| 8 | Postprandial hypotension[Title/Abstract] | 354 |
| 9 | (((Postprandial Period[MeSH Terms]) OR (((((Period*, Postprandial[Title/Abstract]) OR (Postprandial Period*[Title/Abstract])) OR (Postcibal Period*[Title/Abstract])) OR (Period*, Postcibal[Title/Abstract])) OR (Postprandial state*[Title/Abstract]))) AND ((Hypotension[MeSH Terms]) OR ((((Vascular Hypotension[Title/Abstract]) OR (Low Blood Pressure[Title/Abstract])) OR (Blood Pressure, Low[Title/Abstract])) OR (Hypotension, Vascular[Title/Abstract])))) OR (Postprandial hypotension[Title/Abstract]) | 393 |

**Table S2. JBI critical appraisal checklist for studies reporting prevalence data**

| Author, year | Q1 | Q2 | Q3 | Q4 | Q5 | Q6 | Q7 | Q8 | Q9 | AR (%) |
| --- | --- | --- | --- | --- | --- | --- | --- | --- | --- | --- |
| Jang, A.,2020 | Y | N | N | U | Y | N | U | Y | Y | 44.4 |
| Schoevaerdts et al, 2019 | Y | N | N | N | Y | N | N | Y | Y | 44.4 |
| Abbas et al, 2018 | Y | N | N | N | Y | N | N | Y | Y | 44.4 |
| Asensio et al, 2015 | Y | N | N | N | Y | N | N | Y | Y | 44.4 |
| Trahair et al, 2015 | Y | N | N | U | N | N | U | Y | N | 22.2 |
| Tabara et al, 2014 | Y | N | Y | U | Y | N | N | Y | Y | 55.6 |
| Son et al,2012 | Y | N | N | U | Y | N | N | N | Y | 33.3 |
| Fisher et al, 2005 | Y | N | N | U | Y | N | N | Y | Y | 44.4 |
| Vloet et al, 2005 | Y | N | N | Y | Y | N | U | Y | Y | 55.6 |
| Puisieux et al, 2002 | Y | N | N | U | Y | N | U | Y | Y | 44.4 |
| Teramoto et al, 1997 | Y | N | N | N | Y | N | U | Y | Y | 44.4 |
| Aronow et al, 1994 | Y | N | Y | N | Y | N | N | Y | Y | 55.6 |
| Vaitkevicius et al, 1991 | Y | N | N | U | Y | N | N | Y | Y | 44.4 |
| AR (%) | 100 | 0 | 15.4 | 7.7 | 92.3 | 0 | 0 | 92.3 | 92.3 |  |

AR: Adherence rate, N-No, Y-Yes, U-Unclear, NA-Not applicable.

Q1 Was the sample frame appropriate to address the target population?

Q2 Were study participants sampled in an appropriate way?

Q3 Was the sample size adequate?

Q4 Were the study subjects and the setting described in detail?

Q5 Was the data analysis conducted with sufficient coverage of the identified sample?

Q6 Were valid methods used for the identification of the condition?

Q7 Was the condition measured in a standard, reliable way for all participants? Q8 Was there appropriate statistical analysis?

Q9 Was the response rate adequate, and if not, was the low response rate managed appropriately?

Note: The reason for the “N” response for Q6 was that an international consensus on how to diagnose postprandial hypotension is currently lacking.

The study conducted by son et al did not report the 95% confidence interval (CI) for the results; therefore, the answer for Q8 was “No”.

**Table S3. Preparations for diagnosing postprandial hypotension**

| Author, year | Duration of fast | Prohibition  of caffeine | Prohibition  of alcohol | No smoking |
| --- | --- | --- | --- | --- |
| Jang, A., 2020 | 4h | Y(4h) | Y(4h) | Y(4h) |
| Schoevaerdts et al, 2019 | NA | U | U | U |
| Abbas et al, 2018 | NA | U | U | U |
| Asensio et al, 2015 | NA | N | U | U |
| Trahair et al, 2015 | Overnight | U | U | U |
| Tabara et al, 2014 | NA | N | U | U |
| Son et al,2012 | NA | U | U | NA |
| Fisher et al, 2005 | Overnight | U | U | U |
| Vloet et al, 2005 | Overnight | U | U | U |
| Puisieux et al, 2002 | Midnight | N | U | U |
| Teramoto et al, 1997 | Overnight | U | U | NA |
| Aronow et al, 1994 | NA | Y (During the study) | Y (During the study) | Y (During the study) |
| Vaitkevicius et al, 1991 | NA | U | U | U |

NA-not applicable, h-hour, U-unreported, N-no, Y-yes.

**Table S4. Standard test meal**

| Author, year | Contents | Heat quantity | Eating time |
| --- | --- | --- | --- |
| Jang, A., 2020 | 210 g rice, 100 g soup, and 70 g side dishes | 500 kcal | U |
| Trahair et al, 2015 | 300 mL water containing 75 g glucose | 300 Kcal | within 3 min |
| Vloet et al, 2005 | Liquid test meal consisted of a mixture of 100 ml of liquid  glucose-syrup and 100 ml of lactose-free whole milk, containing a total amount of 65 g carbohydrate, 2 g fat, and 4 g protein. | U* | within 10 min |
| Puisieux et al, 2002 | Hot meal consisted of coffee, milk, bread, marmalade, and  natural yoghurt, containing 85.1 g (75%) carbohydrate, 20.7 g (18%) protein, 3.8 g (7%) fat, 409 mg sodium, and 875 mg potassium. | 457 Kcal | within 15 min |
| Teramoto et al, 1997 | 70% carbohydrates, 15% fat, 15% protein, and no caffeine | 700 Kcal | within 15 min |

g-gram, U-unreported, min-minutes, U*-unclear


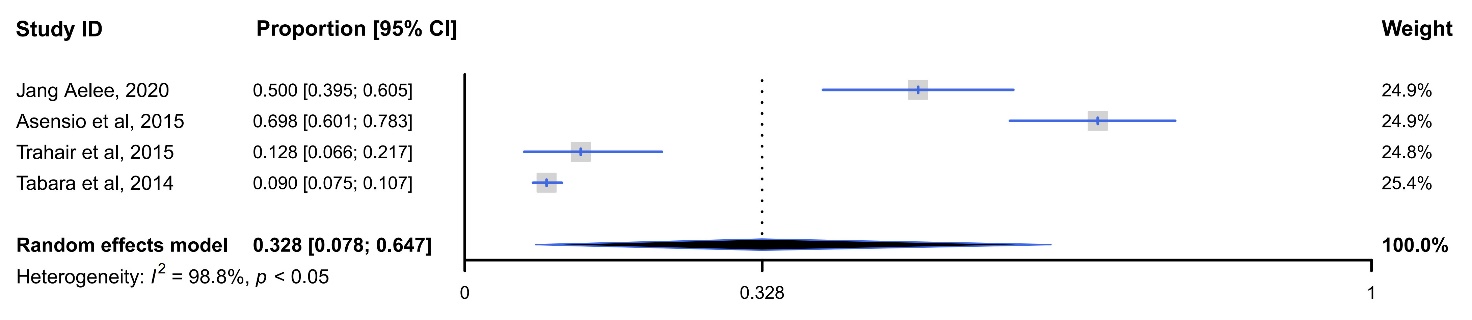


Fig.S1 Forest plot of studies reporting the prevalence of postprandial hypotension in community-dwelling older adults


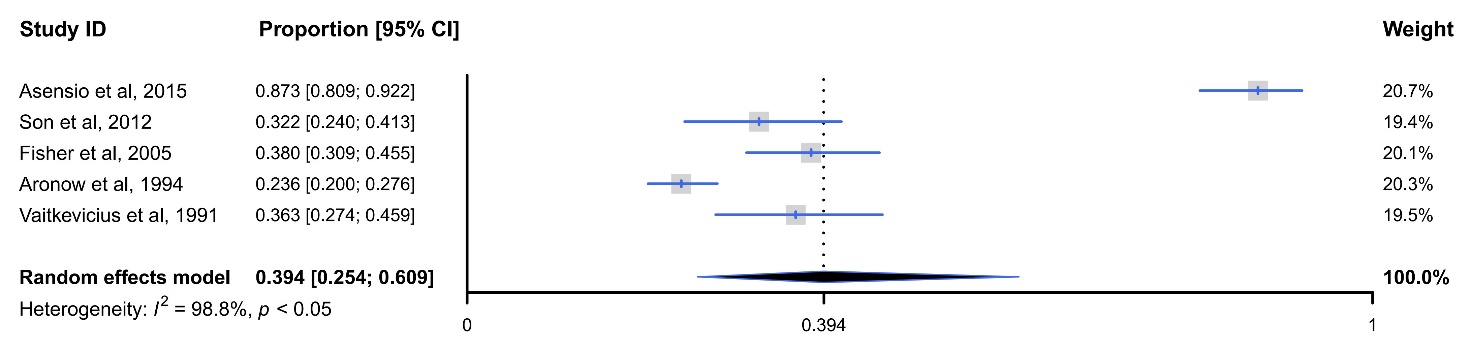


Fig.S2 Forest plot of studies reporting the prevalence of postprandial hypotension in long-term healthcare facility residents


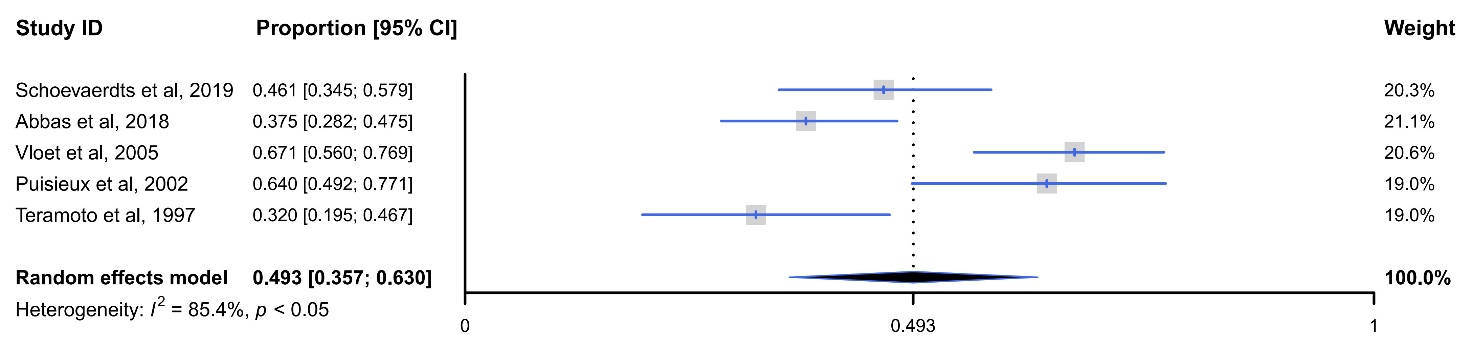


Fig.S3 Forest plot of studies reporting the prevalence of postprandial hypotension in geriatric patients


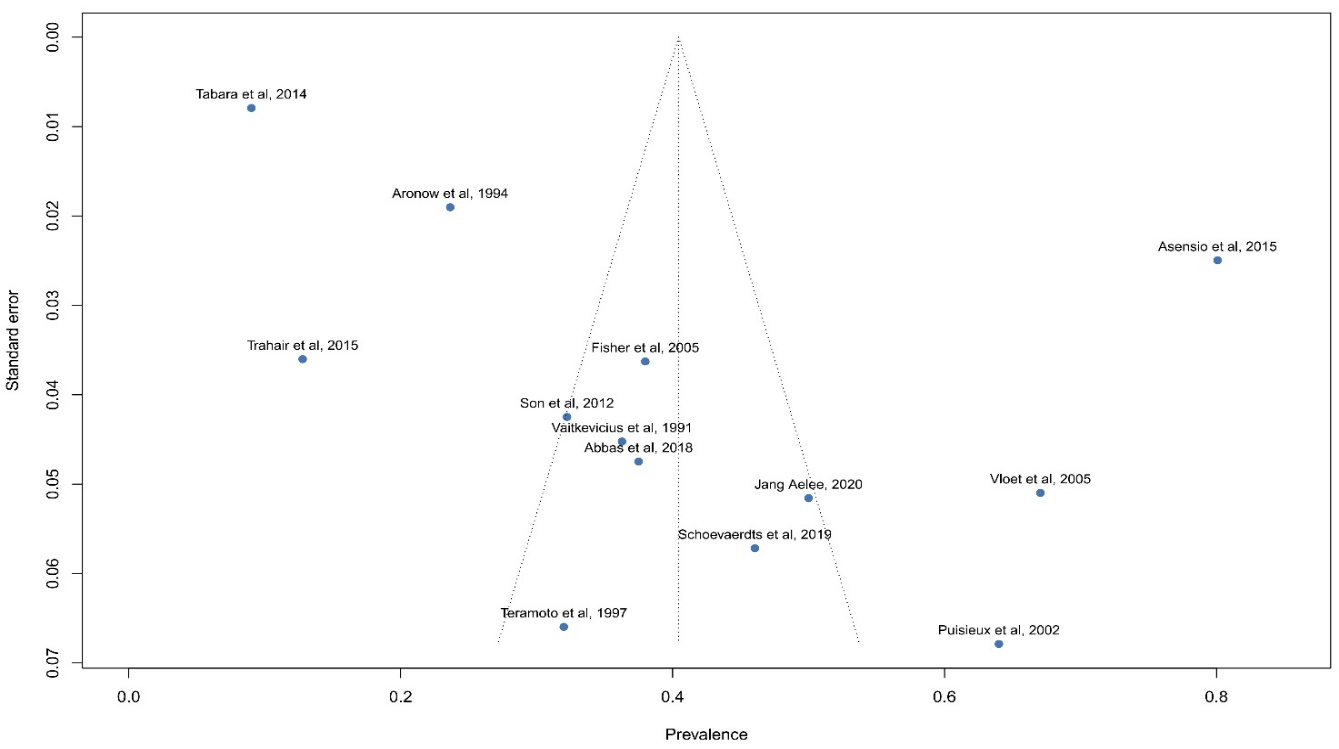


Fig.S4 Funnel plot of studies reporting the prevalence of postprandial hypotension in older adults

**Table S5. Results of univariate meta-regression analysis**

| Covariates | Number of  Studies | Beta (95% CI) | P value | Adj. R2  (%) |
| --- | --- | --- | --- | --- |
| Setting (Ref. Community) | 13 | — | — | 50.8 |
| *Geriatrics department of hospitals | — | 0.262 (0.045–0.478) | 0.017 | — |
| *Retirement homes and communities | — | 0.569 (0.237–0.902) | 0.001 | — |
| long-term healthcare facilities | — | 0.093 (-0.130–0.316) | 0.415 | — |
| Type of diagnostic criteria for PPH (Ref. Type I) | 13 | — | — | 24.7 |
| Type II | — | 0.099 (-0.284–0.483) | 0.611 | — |
| *Type III | — | 0.440 (0.070–0.809) | 0.020 | — |
| #Mean age | 12 | 0.019 (-0.002–0.039) | 0.076 | 16.9 |
| Female (%) | 12 | 0.521 (-0.651–1.692) | 0.384 | 0.0 |
| Hypertension (%) | 11 | 0.064 (-0.497–0.625) | 0.823 | 0.0 |

CI: confidence interval, Adj-adjust, Ref.-reference, PPH-postprandial hypotension. *: P<0.05, Type I-PPH was deﬁned as a decrease in SBP of≥20 mmHg within 120 min after eating; Type II- PPH was defined as a drop of SBP of＞20 mmHg or postprandial SBP ≤ 90 mmHg if pre-prandial SBP was ≥ 100 mmHg within 120 min after eating; Type III- PPH was defined as a reduction of≥20 mmHg for SBP and≥10 mmHg for DBP within 90 min after eating, in both cases compared to pre-prandial BP, # P＞0.05.

**Table S6. Method of diagnosing symptomatic postprandial hypotension**

| Author, year | Evaluation method | Tool | Validated |
| --- | --- | --- | --- |
| Schoevaerdts et al, 2019 | Researchers checked for specific symptoms, such as vertigo, dizziness, nausea, general weakness, thoracic pain, visual complaints and  altered level of conscientiousness during the measurement of postprandial BP. | N | NA |
| Abbas et al, 2018 | A nurse collected clinical manifestations suggesting PPH (syncope, falls, dizziness, weakness, light-headedness, scotoma, nausea, elocution disorder, coronary event, and stroke) during the measurement of postprandial BP. | N | NA |
| Vloet et al, 2005 | ①Symptoms after meal ingestion of participants were observed continuously by the researcher, unaware of the hemodynamic changes during the measurement of postprandial BP.  ②Patient self-report: Participants were asked for symptoms every 15 min | ①Items: N  ②Evaluation results: Symptoms were classiﬁed and coded in a 4-point scale of severity; 0=absence of symptoms, 1=mild, 2 =moderate, and 3 =severe. | N |

N-no, NA-not applicable, PPH-postprandial hypotension, BP-blood pressure, min-minutes
